# Maternal chorioamnionitis and neurodevelopmental outcomes in preterm and very preterm neonates: A meta-analysis

**DOI:** 10.1371/journal.pone.0208302

**Published:** 2018-12-11

**Authors:** Dongqiong Xiao, Tingting Zhu, Yi Qu, Xiaoyun Gou, Qun Huang, Xihong Li, Dezhi Mu

**Affiliations:** 1 Department of Pediatrics, West China Second University Hospital, Sichuan University, Chengdu, China; 2 Key Laboratory of Birth Defects and Related Diseases of Women and Children (Sichuan University), Ministry of Education, Chengdu, China; University of Missouri Columbia, UNITED STATES

## Abstract

**Context:**

No consensus exists regarding the association between maternal chorioamnionitis and neurodevelopmental outcomes in preterm and very preterm neonates.

**Objectives:**

To investigate whether maternal chorioamnionitis affects neurodevelopmental outcomes and to identify the factors that may explain these effects.

**Data sources:**

We used Ovid Medline, EMBASE and Web of Science to conduct a meta-analysis of studies published in English before August 25, 2017, with titles or abstracts that discussed an association between maternal chorioamnionitis and mental/motor development.

**Study selection:**

Among the 603 initially identified studies, we selected those that addressed an association between maternal chorioamnionitis and mental/motor development according to our preselected inclusion criteria as follows: (1) the study compared infants with and without exposure to maternal chorioamnionitis and (2) the neurodevelopmental outcome was followed up using the Bayley Scales of Infant Development 2^nd^ edition.

**Data synthesis:**

Our meta-analysis included 10 studies. According to a random effect model, infants with maternal chorioamnionitis exposure had poorer mental development (d = -2.25 [95%CI, -4.33, -0.17], p<0.05) than infants without maternal chorioamnionitis, and infants with maternal clinical chorioamnionitis exposure had poorer motor development (d = -2.37 [95%CI, -4.62 to -0.12], p<0.05) than infants without maternal clinical chorioamnionitis exposure. Factors in the meta-analysis that showed differences between the two patient groups included an MDI assessment blinded to medical history, MDI assessment at the correct age, and time of the MDI assessment.

**Conclusion:**

This study suggests that maternal chorioamnionitis may affect mental development in preterm and very preterm neonates, and that maternal clinical chorioamnionitis may affect motor development in offspring. Further studies are required to confirm these results and to detect the influence of variables across studies.

## Introduction

Despite advances in obstetric and neonatal care, the prevalence of children with adverse neurodevelopmental outcomes has increased [1], and the etiology of adverse neurodevelopmental outcomes remains poorly understood. Evidence shows that neonatal factors, including premature birth, very low birth weight, necrotizing enterocolitis (NEC), meningitis [2, 3], birth asphyxia, bronchopulmonary dysplasia (BPD) [4, 5], and periventricular-intraventricular hemorrhage (PV-IVH) [6], contribute to adverse neurodevelopmental outcomes. In addition, some prenatal factors, including maternal age[7], education [8, 9], obesity [10–13], race [14], and hypertension [15], contribute to adverse neurodevelopmental outcomes.

Maternal chorioamnionitis is categorized as histologic, microbiologic, and clinical [2]. Maternal histologic chorioamnionitis is defined as pathologic findings on placental histology, including neutrophil infiltration of placental membranes, funisitis (inflammation of the umbilical cord), or fetal vasculitis [16, 17]. Wu et al. characterized maternal microbiological chorioamnionitis as retrieval of microbial organisms in amniotic fluid or placental cultures. Maternal clinical chorioamnionitis is characterized by maternal fever, malodorous amniotic fluid, uterine tenderness, maternal or fetal tachycardia, and maternal leukocytosis [2]. Several studies have shown evidence of an association between maternal infection/chorioamnionitis and neurodevelopmental outcomes [18–21]. This meta-analysis showed that maternal chorioamnionitis was associated with periventricular leukomalacia (PVL) and cerebral palsy (CP) [2]. The relationship between maternal chorioamnionitis and neurodevelopmental outcomes in children has become a topic of increased focus and inconsistency in recent years [3].

To date, many studies have focused on the relationship between maternal chorioamnionitis and neurodevelopmental outcomes. The findings from human studies of maternal chorioamnionitis and neurodevelopmental outcomes of children have been inconsistent. Some studies have indicated no difference in the neurodevelopmental outcomes between infants with and without maternal chorioamnionitis exposure [22–25]. The reason for this finding may be a higher frequency of IVH in infants without maternal chorioamnionitis exposure than in those with material chorioamnionitis exposure. Subsequently, at 7 months of age [22], preterm infants without maternal chorioamnionitis exposure underwent other events that affected neurodevelopmental outcomes [25]. In those studies, the examiners were blinded to the medical histories of the patients and assessed the Mental Development Index (MDI) or Psychomotor Development Index (PDI) scores of these infants [23, 24], while some studies assessed the MDI or PDI of infants after correction for gestational age [23, 24]. However, some studies have indicated a significant difference in the neurodevelopmental outcomes between infants with and without maternal chorioamnionitis exposure [26, 27]. Thus, we performed a meta-analysis to evaluate this association.

## Methods

### Retrieval of studies

The Ovid Medline, EMBASE, and Web of Science databases were searched through August 25, 2017. The search for mental/motor development was performed using the following keywords and subject terms: “Bayley scales”, or “Bayley*”, or “BSID”, or “neurodevelopment”, or “neuropsycholog*”, or “child development”, or “executive functioning”, or “intelligence”, or “psychomotor”, or “aptitude test”, using “OR” to link relevant text within the search field. To acquire studies related to chorioamnionitis, “OR” was used to associate the key words, which included “chorioamnionitis”, “intraamniotic”, and “intra-amniotic”. We combined these terms using “AND” to retrieve the studies. We restricted the search to human studies published in English. The retrieved studies were screened by reading the titles and abstracts, and two authors (Dongqiong Xiao and Tingting Zhu) subsequently read the full text of the remaining publications independently and then discussed disagreements to reach a consensus.

### Study selection

The study inclusion criteria were as follows: (1) the study compared infants with and without maternal chorioamnionitis exposure; (2) the study was published in English; (3) the neurodevelopmental outcome assessment was measured using only the Bayley Scales of Infant Development 2^nd^ edition (BSID-II) [28], which is the most commonly used scale to assess neurodevelopmental outcomes [29]; (4) the scale consisted of the PDI and MDI; and (5) the study reported the PDI and MDI scores with a mean (standard deviation, SD) of 100 (15). Lower scores indicated poorer neurodevelopmental outcomes.

The exclusion criteria for the study were as follows: (1) the study was a review or meta-analysis; (2) the study was not published in English; (3) the study was a case report; (4) the article described an animal experiment study; (5) study had data that overlapped with that of another study; (6) the study did not report the children’s PDI and MDI scores with and without maternal chorioamnionitis exposure, and (7) the study did not have useable data.

### Data extraction

The data were independently extracted from the studies by two reviewers (Dongqiong Xiao and Tingting Zhu) and included the name of the first author, publication year, number of cases in the exposure and control groups, primary outcome (MDI or PDI), gestational age, and category of maternal chorioamnionitis.

### Quality evaluation

The two reviewers (Dongqiong Xiao and Tingting Zhu) independently used the Newcastle-Ottawa Scale (NOS) [30] to assess each of the included studies for its methodological quality. The reviewers evaluated the quality score by assessing the selection of the study population (four items), comparability (one item), and evaluation of exposure and outcome (three items). Disagreements were resolved in the manner previously described.

### Statistical analysis

The original studies included used the mean and SD to assess the MDI and PDI of the infants. We pooled the MDI and PDI scores of each study separately using the DerSimonian-Laird formula (random-effects model) [31]. Statistical heterogeneity [32] between the studies was assessed using the Q and *I*^*2*^ statistics. Values of p<0.1 and *I*^*2*^>50% indicated high heterogeneity [2]. We conducted a subgroup analysis based on the type of chorioamnionitis (clinical or histologic) as well as a stratified analysis of the effect of maternal chorioamnionitis on the MDI of the infants based on the blinding of the medical history in the MDI assessment (blinded or not blinded), the MDI assessment after correction for gestational age (the MDI of the infants assessed at the correct age or the MDI of the infants assessed at an incorrect age), and the time of the MDI assessment (at 7 months, 12 months, or 18–24 months). We performed sensitivity analyses by omitting one study at a time.

We used a funnel plot to assess the publication bias. We used Egger’s [33] and Begg’s [34] tests to assess the publication bias, which was considered to be statistically significant when p<0.05. We performed the statistical tests using Stata software, version 12.0 (StataCorp, College Station, TX) and Review Manager 5.3.

## Results

### Literature search

**Fig 1:**
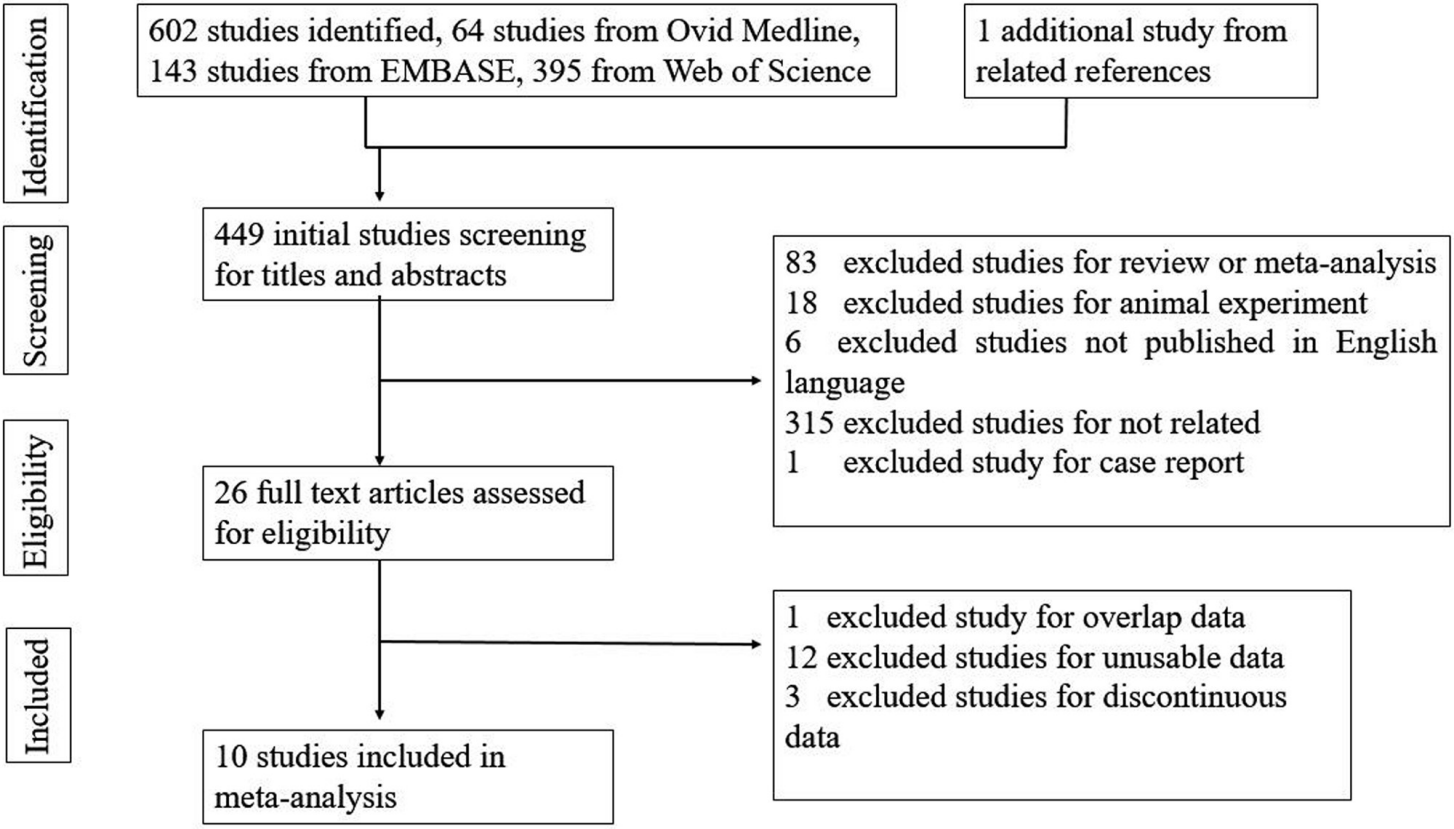
Flow chart for study selection.

We identified 603 potential studies, with 64 from Ovid Medline, 143 from EMBASE, 395 from Web of Science, and 1 study from a related reference (S1 Data). Following the removal of duplicates, 449 studies remained; these were screened for inclusion in the review. Of these, 423 studies were excluded due to being reviews, meta-analyses, animal experiments, case reports, not relevant to the subject, ort not published in English, leaving 26 full texts to be assessed for eligibility. Sixteen studies were excluded (see Fig 1 and the excluded studies with the reasons for exclusion). After careful screening, 10 studies that reported BSID-II scores were selected for inclusion in this study (see Fig 1). These 10 included studies are summarized in Table 1.

**Table 1:** Characteristics of the included studies.

Study	Publication year	No. of cases in exposure/control groups	Primary outcome	Gestational age	MDI assessment	Exposure to chorioamnionitis
Watterberg	2007	55/18	MDI, PDI	<28 w	Masked; at correct age; 18 m	Histologic
Schlapbach	2010	33/33	MDI, PDI	<32 w	Masking not mentioned; at correct age; 24 m	Clinical
Vander Haar	2016	194/1380	MDI, PDI	<32 w	Masking not mentioned; not at correct age; 24 m	Clinical
Hardt	1985	42/31	MDI, PDI	<32 w	Masking not mentioned; not at correct age; 12 m	Clinical
Morales	1987	43/43	MDI, PDI	<32 w	Masking not mentioned; at correct age; 12 m	Clinical
Mu	2007	54/41	MDI, PDI	<34 w	Masking not mentioned; at correct age; 24 m	Clinical
Polam	2005	102/75	MDI, PDI	<29 w	Masked; at correct age; 24 m	Histologic
Kaukola	2006	24/29	MDI	<32 w	Masked; at correct age; 24 m	Histologic
Dexter	2000	100/67	MDI, PDI	<28 w	Masking not mentioned; at correct age; 7 m	Clinical
Hendson	2011	229/271	MDI	<32 w	Masking not mentioned; not at correct age; 18 m	Histologic

### Characteristics and quality of the included studies

The included studies were published between 1985 and 2011. Ten studies [20, 22–27, 35–37] reported the MDI for infants with maternal chorioamnionitis exposure; six of these studies [20, 22, 26, 35–37] reported maternal clinical chorioamnionitis, and the other four studies [23–25, 27] reported maternal histologic chorioamnionitis. A total of 876 infants were exposed to maternal chorioamnionitis, and a total of 1988 infants were not exposed to maternal chorioamnionitis. Eight studies[22, 24–26, 35–38] reported the PDI of infants with maternal chorioamnionitis exposure. These infants were preterm infants. Three studies[23, 24, 37] assessed the MDI with masking of the clinical data, and seven studies[22, 26, 27, 35–38] assessed the MDI without mentioning masking of the clinical data. Seven studies[22–25, 35–37] assessed the MDI at the correct age of the infants, and three studies[26, 27, 38] assessed the MDI without mentioning whether it was performed at the correct age of the infants. One study[22] assessed the MDI of the infants at 7 months, two studies[26, 35] assessed the MDI of the infants at 12 months, two studies[25, 27] assessed the MDI of the infants at 18 months, and 5 studies[23, 24, 36–38] assessed the MDI of the infants at 24 months. All the included studies were case-control studies of a high quality (NOS>5) (S2 Data).

### Maternal chorioamnionitis and neurodevelopmental outcomes

#### Mental development

**Fig 2:**
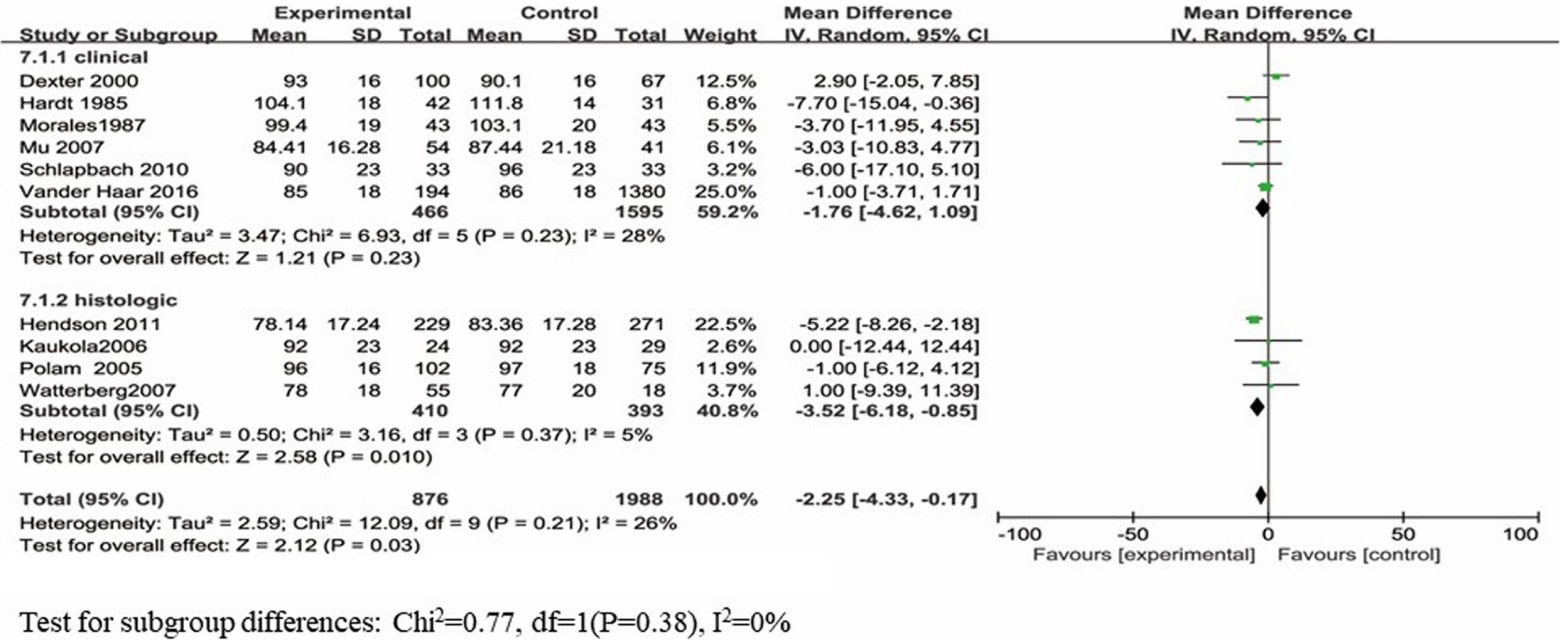
Forest plot of pooled analysis of maternal chorioamnionitis and the MDI in offspring.

The BSID-II MDI scores were reported in 10 studies, which included a total of 2864 infants. Eight of the ten studies reported no significantly poorer MDI scores, and two of the ten studies reported significantly poorer MDI scores for infants with maternal chorioamnionitis exposure than those for infants without maternal chorioamnionitis exposure. When the study results were analyzed using the random-effects model, infants with maternal chorioamnionitis exposure had significantly poorer MDI scores than those without exposure (d = -2.25; 95% CI: -4.33 to -0.17; p<0.05) (Fig 2). The data were heterogeneously distributed (*I*^*2*^ = 26%, p = 0.21).

#### Motor development

**Fig 3:**
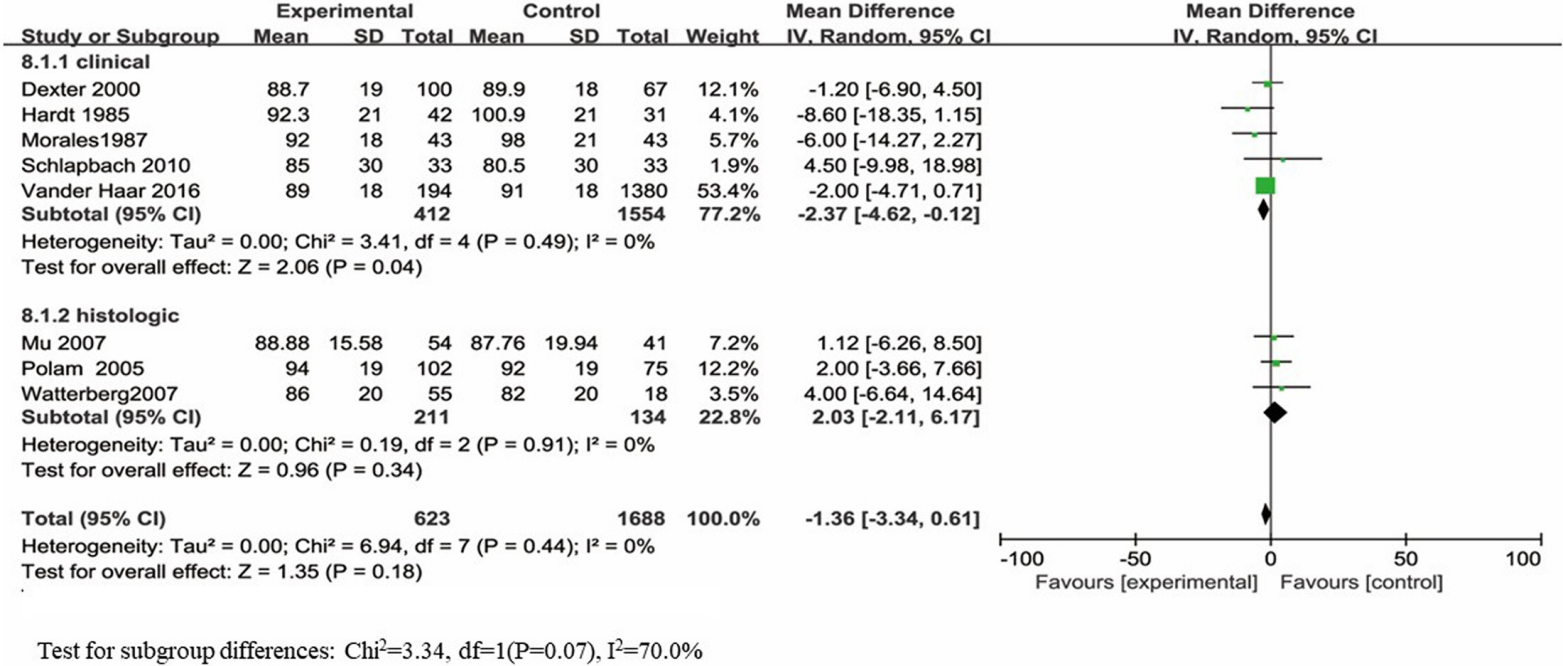
Forest plot of pooled analysis of maternal chorioamnionitis and the PDI in offspring.

The BSID-II PDI scores were reported in 8 studies, which included a total of 2311 infants, and only one study showed significantly poorer PDI scores for infants with maternal chorioamnionitis exposure than for infants without maternal chorioamnionitis exposure. No significant difference was observed in the PDI scores between infants with and without maternal chorioamnionitis exposure (d = -1.36; 95% CI: -3.34 to 0.61; p = 0.18) (Fig 3). The data were heterogeneously distributed (*I*^*2*^ = 0%, p = 0.44).

### Subgroup analysis

The subgroup analysis was based on maternal clinical and histologic chorioamnionitis. Six articles [20, 22, 26, 35–37] reported the MDI scores based on maternal clinical chorioamnionitis exposure, and four articles [23–25, 27] reported the MDI scores based on maternal histologic chorioamnionitis exposure.

No difference was observed in the MDI scores between infants with and without maternal clinical chorioamnionitis exposure (d = -1.76; 95% CI: -4.62 to 1.09; p = 0.23). These data were heterogeneously distributed (*I*^*2*^ = 28%, p = 0.23). Infants with maternal histologic chorioamnionitis exposure had significantly poorer MDI scores than controls (d = -3.52; 95% CI: -6.18 to -0.85; p = 0.01). These data were heterogeneously distributed (*I*^*2*^ = 0%, p = 0.37).

Five articles [20, 22, 26, 35, 37] reported the PDI scores based on maternal clinical chorioamnionitis exposure. A significant difference was observed in the PDI scores between the infants with and without maternal clinical chorioamnionitis exposure (d = -2.37; 95% CI: -4.62 to -0.12; p = 0.04), and the data were heterogeneously distributed (*I*^*2*^ = 0%, p = 0.49). Three [24, 25, 36] studies evaluated the differences in PDI scores between infants with and without maternal histologic chorioamnionitis exposure; these studies showed no significant difference (d = 2.03; 95% CI: -2.11 to 6.17; p = 0.34), and the data were heterogeneously distributed (*I*^*2*^ = 0%, p = 0.91).

A separate meta-analysis was performed after stratification by the factors used in the statistical analysis. Table 2 summarizes the effect of maternal chorioamnionitis on the MDI of infants.

**Table 2:** Pooled results of the associations between maternal chorioamnionitis and the mental development outcome MDI.

Variables	Studies (n)	Mean difference (95% CI)	*I*^*2*^ (p-value for heterogeneity)
Total	10	-2.55 (-4.33, -0.17)	26% (0.21)
Type of chorioamnionitis			
Clinical chorioamnionitis	6	-1.76 (-4.62, 1.09)	28% (0.23)
Histologic chorioamnionitis	4	-3.52 (-6.18, -0.85)	5% (0.37)
Mental development outcome of the MDI assessment			
Infants’ correct age	7	-0.47 (-3.21, 2.26)	0% (0.70)
Did not mention the infants’ correct age	3	-3.86 (-7.57, -0.15)	65% (0.06)
Mental development outcome of the MDI assessment			
18–24 months	7	-2.57 (-4.32, -0.81)	0% (0.47)
12 months	2	-5.93 (-11.41, -0.45)	0% (0.48)
7 months	1	2.90 (-2.05, 7.85)	NA
Mental development outcome of the MDI assessment			
Masked	3	-0.54 (-4.85, 3.77)	0% (0.94)
Without masking	7	-2.74 (-5.48, 0.00)	47% (0.08)

NA: not available.

### Sensitivity analysis

Regarding maternal clinical chorioamnionitis, when the study by Dexter[22] was omitted, the heterogeneity decreased (*I*^*2*^ = 0%, p = 0.47), and regarding maternal histologic chorioamnionitis, when the study by Polam[24] was omitted, the heterogeneity also decreased (*I*^*2*^ = 0%, p = 0.45).

### Publication bias

**Fig 4:**
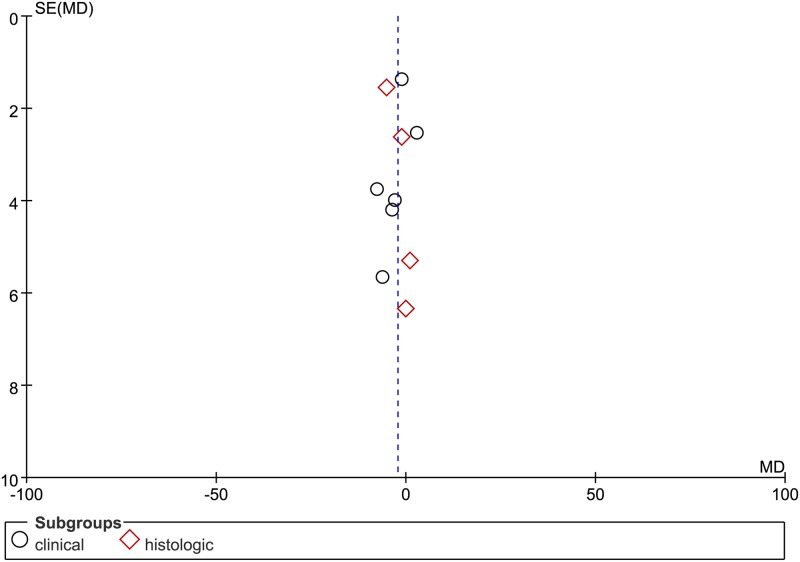
The funnel plot for publication bias test. Funnel plot with pseudo 95% CI of MDI.

**Fig 5:**
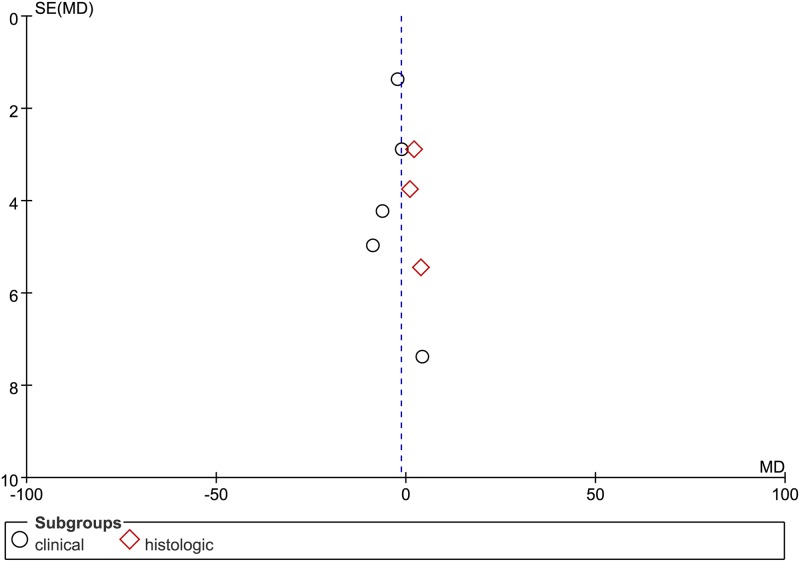
The funnel plot for publication bias test. Funnel plot with pseudo 95% CI of PDI.

Funnel plots were used to assess the potential publication bias (see Figs 4 and 5). The pooled results showed no evidence of significant publication bias.

## Discussion

To our knowledge, van Vliet et al.[3] have conducted a meta-analysis of the association between perinatal infection and neurodevelopmental outcomes in very preterm neonates, we have focused on the association between maternal chorioamnionitis and neurodevelopmental outcomes in preterm and very preterm neonates. The results of this meta-analysis, which included 10 studies, showed evidence that infants with maternal chorioamnionitis exposure have a poorer MDI than those without maternal chorioamnionitis exposure. Additionally, 8 studies showed no significant difference in the PDI scores between offspring with and without maternal chorioamnionitis exposure.

Maternal chorioamnionitis might affect the neurodevelopment of infants through multiple pathways. Human data studies show that chorioamnionitis contributes to CP [2, 39], NEC [40], premature birth, low 5-minute Apgar scores[22], and BPD[41]. Additionally, other studies of human data show that NEC [3], premature birth, low 5-minute Apgar scores, and BPD[4] contribute to adverse neurodevelopmental outcomes; these studies indirectly suggest that chorioamnionitis may contribute to impaired neurodevelopmental outcomes. Berger et al. showed that infants with positive maternal amniotic cavity cultures had a significantly higher risk of an adverse PDI score [1]. Dammann et al. showed that perinatal infection does indeed contribute to long-term cognitive deficits in offspring[42]. Mir et al. showed that placental inflammatory villitis contributes to abnormal neurodevelopmental outcomes[43]. Additionally, many studies have shown that maternal chorioamnionitis contributes to adverse neurodevelopmental outcomes [16, 21, 44, 45].

Evidence from animal models shows that maternal chorioamnionitis affects brain development. Maternal chorioamnionitis may result in infection-mediated fetal brain injury and epigenetic changes[46], thus altering neurodevelopment outcomes later in life[18]. Proinflammatory cytokines produced by inflammatory cells can cause neurotoxicity, oligodendrocyte maturation arrest or injury, disruption of myelination and demyelination, and microglia activation [47–52]. These brain cell dysfunctions may result in impaired neurodevelopmental outcomes. Chorioamnionitis is closely related to fetal white matter injury, thus resulting in neurodevelopmental complications [6], including autism, cognitive impairments and CP [53]. This may explain the effects of maternal chorioamnionitis on mental development. Maternal chorioamnionitis may contribute to maladaptive programming of the fetal brain [8].

To better understand the effect of maternal chorioamnionitis on the MDI of infants, we divided the maternal chorioamnionitis group into clinical chorioamnionitis and histologic chorioamnionitis groups. Maternal histologic chorioamnionitis impairs mental development. Regarding maternal clinical chorioamnionitis, when the study by Dexter [22] was omitted from the meta-analysis, the heterogeneity decreased to 0%, and the meta-analysis showed that maternal clinical chorioamnionitis significantly impaired mental development. Therefore, the heterogeneity is mainly due to the study by Dexter [22].

However, the views on this topic are inconsistent. Hendson et al. showed that maternal histologic chorioamnionitis had no significant effect on neurodevelopmental impairment after adjustment for perinatal variables [27]. In the subgroup analysis, maternal clinical chorioamnionitis had no effect on mental development, and maternal histologic chorioamnionitis had no effect on motor development. There are several possible reasons for these contradictory results. The definitions of clinical chorioamnionitis and histologic chorioamnionitis are inconsistent. Some published articles do not use a specific diagnostic definition for maternal clinical chorioamnionitis, and these articles produce heterogeneous and contradictory results. In the separated stratified meta-analysis, the common correction for gestational age may underestimate the contribution of maternal chorioamnionitis to the neurodevelopmental outcomes of infants. The varied timing of MDI assessments of infants may affect the results regarding the contribution of maternal chorioamnionitis to the neurodevelopmental outcomes of the infants, such as in the study by Dexter [22] that assessed the MDI at 7 months, and this is the major source of heterogeneity for this meta-analysis. Furthermore, preterm birth in the control group without maternal chorioamnionitis exposure may induce exposure to other devastating events (i.e., IVH, cystic PVL, etc.) that can affect neurodevelopmental outcomes [22]. When the examiners are not blinded to the medical history, the MDI or PDI assessments of the infants may overestimate the contribution of maternal chorioamnionitis to the neurodevelopmental outcomes. This demonstrates the importance of using a blinded examination when assessing the mental and motor development outcomes of infants.

To confirm that maternal chorioamnionitis could independently contribute to neurodevelopmental impairment, future studies should consider additional potential confounding variables. Maternal chorioamnionitis may affect neurodevelopment due to factors such as preterm birth, the criteria used to define chorioamnionitis, a lack of masking of the medical history at the MDI assessment of infants, MDI assessment of infants at the correct age, the timing of the MDI assessment and other potential cofounding variables. Although the meta-analysis considered several confounding variables, the contribution of potential biases due to other factors to the neurodevelopmental impairment of children cannot be excluded. Publication bias and an incomplete ascertainment of the published articles may have occurred. We did not find significant evidence for the presence of publication bias in the current meta-analysis.

One shortcoming of our meta-analysis is that the availability of published articles on this subject is currently limited, and the current work partly relies on past study of van Vilet et al.[3]. Additionally, we only included articles published in English. Therefore, the results of this meta-analysis should be interpreted with caution because the number of studies included is small. Furthermore, the BSID-II, which is the most widely used instrument to assess neurodevelopment [29, 54], does have some restrictions, including that it consists of subjective observations and classifications by examiners when assessing the mental and motor performances, the examiners are not blinded to medical history in some studies [20, 22, 26, 35–37], and it has poor predictivity for neurodevelopmental outcomes in children of a school age [29]. The strict inclusion criteria of this meta-analysis may have excluded some studies that evaluated the association between maternal chorioamnionitis and adverse neurodevelopmental outcomes in offspring.

One of the major advantages of our study is that the source of heterogeneity was clear, which overcomes the limitation of the small sample size that limits most articles in determining the effects of maternal chorioamnionitis on the MDI of infants.

In conclusion, our pooled analyses provide evidence that maternal chorioamnionitis may affect mental development (MDI) and maternal clinical chorioamnionitis may affect motor development (PDI) in offspring. Future studies that consider additional factors are required to resolve this issue.

## Supporting information

S1 Checklist PRISMA checklist. (DOC)

S1 Data Retrieval strategy. (DOCX)

S2 Data New Castle Ottawa(NOS) quality assessment for included studies. (DOC)
