# Supplementary material for: Maternal chorioamnionitis and neurodevelopmental outcomes in preterm and very preterm neonates: A meta-analysis
Source: PLoS One. 2018 Dec 11;13(12):e0208302. doi: 10.1371/journal.pone.0208302 (PMC6289416; doi:10.1371/journal.pone.0208302)
Supplement: S1 Data — (DOCX) [file pone.0208302.s002.docx]

**Supplement 1. Retrieval strategy of Ovid Medline, EMBASE and Web of Science**

Before 25 August 2017.

**Ovid Medline**

1 exp chorioamnionitis/

2 chorioamnionitis.ti,ab.

3 ((intraamniotic adj3 infect*) or (intra-amniotic adj3 infect*)).ti,ab.

4 1 or 2 or 3

5 exp intelligence/

6 exp "Bayley Scales of Infant Development"/

7 exp child development/

8 exp aptitude test/

9 exp psychomotor disorder/ or exp psychomotor development/ or exp psychomotor performance/

10 (bayley scales or BSID or Bayley* or neurodevelopment or neuropsycholog* or child development or executive functioning or intelligence or psychomotor or aptitude test).ti,ab.

11 5 or 6 or 7 or 8 or 9 or 10

12 4 and 11

**EMBASE**

Before 25 August 2017.

1 exp chorioamnionitis/

2 chorioamnionitis.ti,ab.

3 ((intraamniotic adj3 infect*) or (intra-amniotic adj3 infect*)).ti,ab.

4 1 or 2 or 3

5 exp intelligence/

6 exp "Bayley Scales of Infant Development"/

7 exp child development/

8 exp aptitude test/

9 exp psychomotor disorder/ or exp psychomotor development/ or exp psychomotor performance/

10 (bayley scales or BSID or Bayley* or neurodevelopment or neuropsycholog* or child development or executive functioning or intelligence or psychomotor or aptitude test).ti,ab.

11 5 or 6 or 7 or 8 or 9 or 10

12 4 and 11

**Web of science**

Before 25 August 2017.

TS=(Bayley Scales of Infant Development or bayley scales* or BSID or Bayley* or neurodevelopment or neuropsychology* or child development or executive functioning or intelligence or psychomotor or aptitude test)

TS=(CHORIOAMNIONITIS or chorioamnionitis or intraamniotic or intra-amniotic)
