# Supplementary material for: Maternal chorioamnionitis and neurodevelopmental outcomes in preterm and very preterm neonates: A meta-analysis
Source: PLoS One. 2018 Dec 11;13(12):e0208302. doi: 10.1371/journal.pone.0208302 (PMC6289416; doi:10.1371/journal.pone.0208302)
Supplement: S2 Data — (DOC) [file pone.0208302.s003.doc]

**Maternal chorioamnionitis and neurodevelopment outcome in offspring: A Meta-analysis**

**Supplement 2. Newcastle - Ottawa Quality Assessment Scale results for case-control** studies

| Question | **Option** | Dexter^22^ | Hardt^26^ | Hendson^27^ | Schlappach^37^ | Morales^35^ | Vander Haar^20^ | Mu^36^ | Polam^24^ | Watterberg^25^ | Kaukola^23^ |
| --- | --- | --- | --- | --- | --- | --- | --- | --- | --- | --- | --- |
| Is the case definition adequate? | a) yes, with independent validation *****  b) yes, eg record linkage or based on self reports  c) no description | a | a | a | a | a | a | a | a | a | a |
| Representativeness of the cases | a) consecutive or obviously representative series of cases *****  b) potential for selection biases or not stated | b | b | b | b | b | b | b | b | b | b |
| Selection of Controls | a) community controls *****  b) hospital controls  c) no description | b | b | b | b | b | b | b | b | b | b |
| Definition of Controls | a) no history of disease (endpoint)*****  b) no description of source | a | a | a | a | a | a | a | a | a | a |
| Comparability of cases and controls on the basis of the design or analysis | 1. study controls for (Select the most important factor.***** 2. study controls for any additional factor (This   criteria could be modified to indicate specific  control for a second important factor.) ***** | a | a | a | a | a | a | a | a | a | a |
| Ascertainment of exposure | a) secure record (eg surgical records)*****  b) structured interview where blind to case/control status*****  c) interview not blinded to case/control status  d) written self report or medical record only  e) no description | a | a | a | a | a | a | a | a | b | b |
| Same method of ascertainment for cases and controls | a) yes*****  b) no | a | a | a | a | a | a | a | a | a | a |
| Non-Response rate | a) same rate for both groups*****  b) non-respondents described  c) rate different and no designation | a | a | a | a | a | a | a | a | a | a |
